# Navigating Parotid Lymphoepithelial Cysts in HIV Patients: A Tale of Two Distinct Scenarios

**DOI:** 10.7759/cureus.50399

**Published:** 2023-12-12

**Authors:** Aditya S Pedaprolu, Suhas Jajoo, Chandrashekhar Mahakalkar, Shivani Kshirsagar, Suhit Naseri

**Affiliations:** 1 General Surgery, Datta Meghe Institute of Higher Education and Research, Wardha, IND; 2 Pathology, Datta Meghe Institute of Higher Education and Research, Wardha, IND

**Keywords:** benign lymphoepithelial cyst, head and neck swellings, parotid gland, human immunodeficiency virus, lymphoepithelial cyst, diffuse infilterative lymphocytic syndrome

## Abstract

A rare occurrence in HIV-infected individuals is the development of diffuse infiltrative lymphocytic syndrome (DILS), which is marked by a widespread infiltration of CD8+ lymphocytes in body tissues, persistent elevation of CD8+ lymphocyte levels, as well as bilateral parotid swellings and cervical lymphadenopathy. It is distinct due to its suspected autoimmune origin and is found in about 5-10% of people living with HIV.

This retrospective analysis involves two patients admitted to our tertiary care rural hospital with complaints of bilateral parotid swellings, a provisional diagnosis of DILS associated with HIV-positive status and lymphoepithelial cysts, their subsequent management, including conservative treatment and surgical excision of one of our patients. Our goal is to contribute to and advance the knowledge of this rare condition.

## Introduction

A rare occurrence in HIV-infected individuals is the development of diffuse infiltrative lymphocytic syndrome (DILS). There is widespread infiltration of CD8 lymphocytes into various organs, leading to continually elevated levels of CD8 lymphocytes in blood. DILS is characterized by the infiltration of CD8+ T lymphocytes into multiple tissues and organs throughout the body, affecting organs such as the liver, lungs, and gastrointestinal tract. Individuals with DILS often display persistently elevated levels of CD8+ T lymphocytes in their blood. A hallmark clinical feature of DILS is the presence of bilateral parotid gland swelling. Additionally, cervical lymphadenopathy, which refers to the enlargement of lymph nodes in the neck, is a common finding in individuals with DILS [1].

A few lymph nodes become enclosed within the parotid gland during embryonic development, containing the structures of salivary glands. When HIV replicates in the body, it leads to the enlargement of these lymphoid tissues in the salivary glands, which could result in swelling of the parotid glands. Around 40% of HIV-positive individuals experience symptoms or signs related to their head and neck [1]. Lymphoepithelial cyst refers to a benign, locular lesion that affects the head and neck region. It is a benign embryonic-dysplastic cystic tumour that most frequently impacts HIV-positive patients and seldom affects people without AIDS. Upto 10% of HIV-infected individuals experience parotid gland enlargement, and the most prevalent of these lesions, i.e., lymphoepithelial cysts, affect 3-6% of HIV patients and are occasionally the initial sign of HIV infection [2]. They usually present as a slow-growing, painless mass, and the occurrence of any secondary infection may cause acute inflammatory symptoms [3]. These lesions have been found to manifest over salivary glands or associated lymph nodes, and less commonly, in the oral cavity, tonsil, thyroid gland, pancreas, or juxta-bronchial regions [4].

The occurrence of these lesions has decreased since the use of anti-retroviral medications. Any cystic parotid gland enlargement in the HIV-negative population justifies a serological examination for the potential diagnosis of HIV. Histologically, lymphoepithelial cysts typically surround the cystic spaces and appear to develop in the parotid nodes. Typically, it consists of squamous or cuboidal epithelium with germinal centres and a substantial infiltration of lymphoid cells that line the cysts [5]. The differential diagnosis of parotid lymphoepithelial cysts includes Warthin's tumour (monomorphic adenoma of the salivary glands), benign hemangioma, branchial cleft cyst, or lymphoma [6].

These cysts can be solitary or numerous and can grow to enormous sizes. They generally affect the parotid glands' superficial lobes, generally bilaterally, and are painless and soft. Despite being benign, the cysts can result in severe aesthetic defects and gradually grow in size. These cysts do not affect the progression of HIV. Most individuals with benign lymphoepithelial cysts are asymptomatic; thus, aesthetic concerns are the primary motivation for treatment. If left untreated, lymphoepithelial cysts can develop into malignant tumours, affecting the extranodal sides. Repeat aspiration, medical management, anti-retroviral therapy, sclerosing therapy, radiation therapy, and surgery are available treatments for managing lymphoepithelial cysts [7].

## Case presentation

Case one

**Figure 1:**
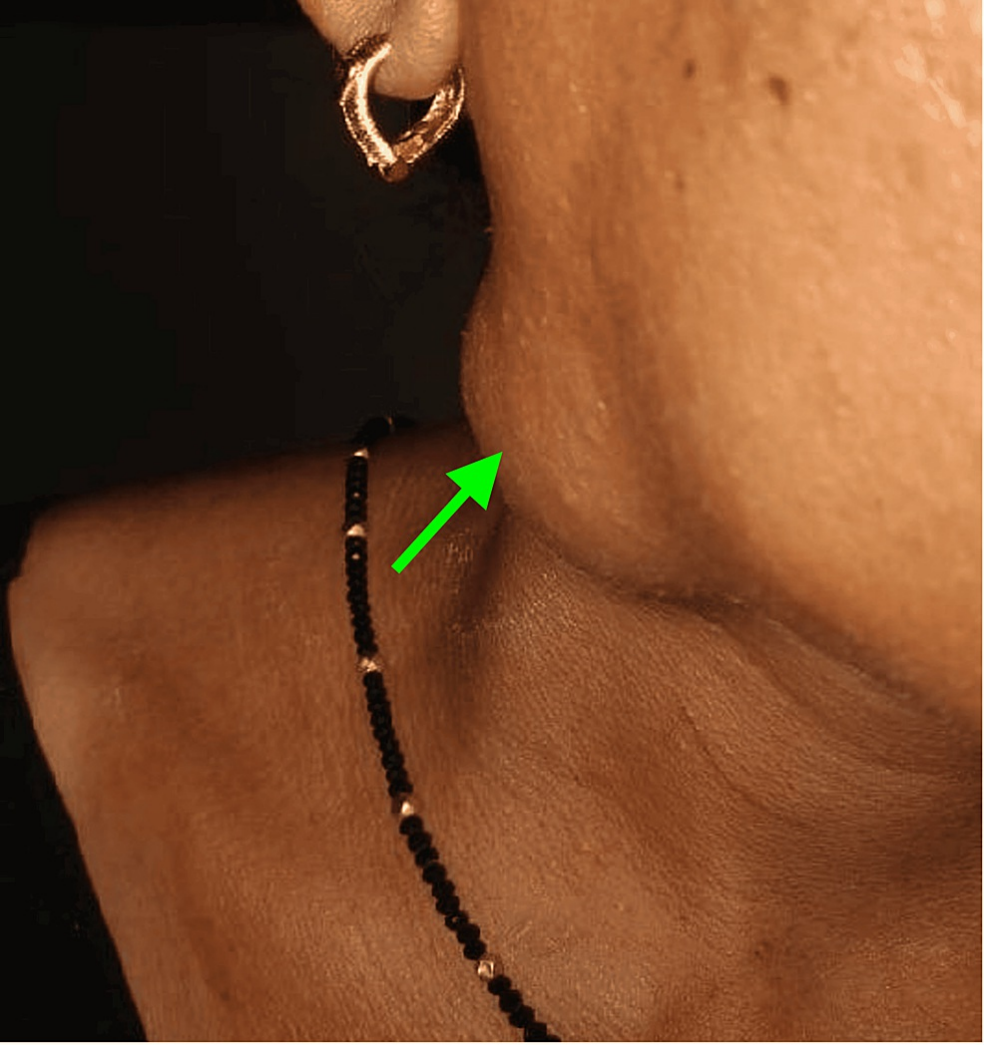
Pre-operative clinical picture Swelling marked with green arrow

A 36-year-old female patient presented to our clinic with chief complaints of swelling over the right side of her face for three years (Figure 1). The swelling was initially small (roughly 2X2 cm) and had later progressed to the current size of approximately 5 x 5 cm. There were no inflammatory changes, pain association, or functional restrictions. However, the patient's main concern was that the swelling was cosmetically evident. There was no evidence of cervical lymphadenopathy.

Ultrasonography-guided fine needle aspiration cytology was done, and 3ml of straw-coloured fluid was aspirated. Smears showed few lymphocytes, histiocytes, and plasma cells correlating the cyst to its lymphoepithelial origin. Routine blood investigations were performed along with an enzyme-linked immunosorbent assay (ELISA) and Western blot test, which were positive. For cosmetic purposes, the patient was willing to undergo surgical excision of the right-sided swelling.

**Figure 2:**
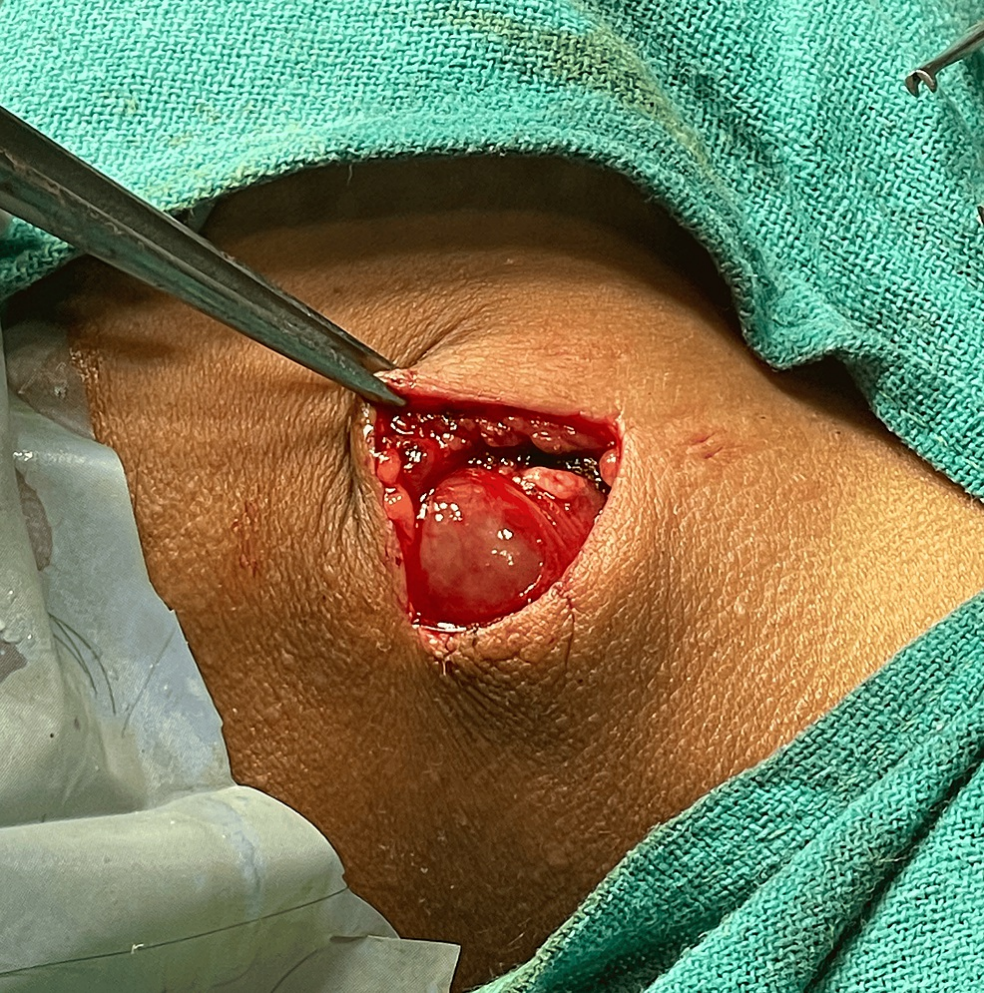
Intra-operative picture taken after identifying the swelling

**Figure 3:**
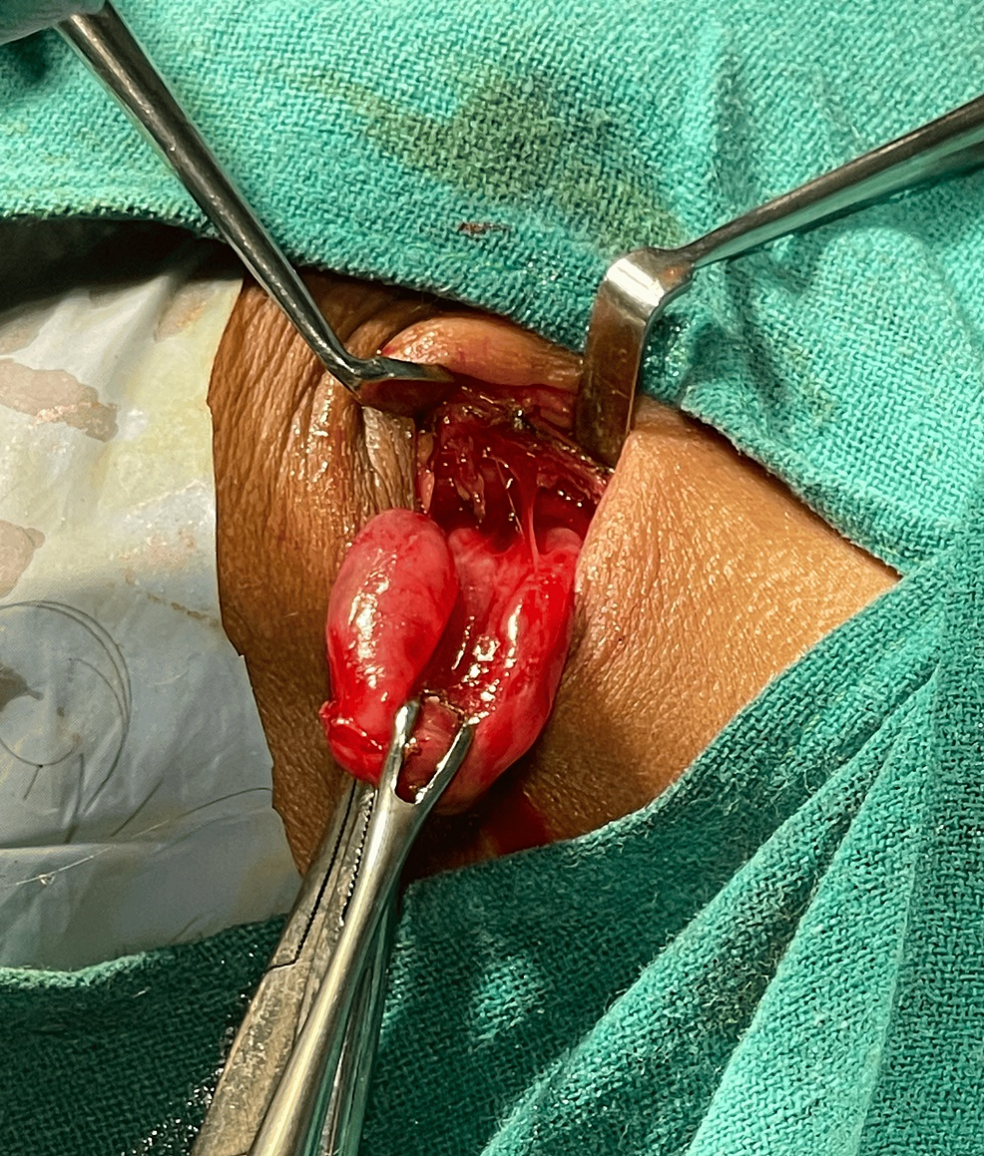
swelling excision after meticulous dissection and separating from surrounding structures

**Figure 4:**
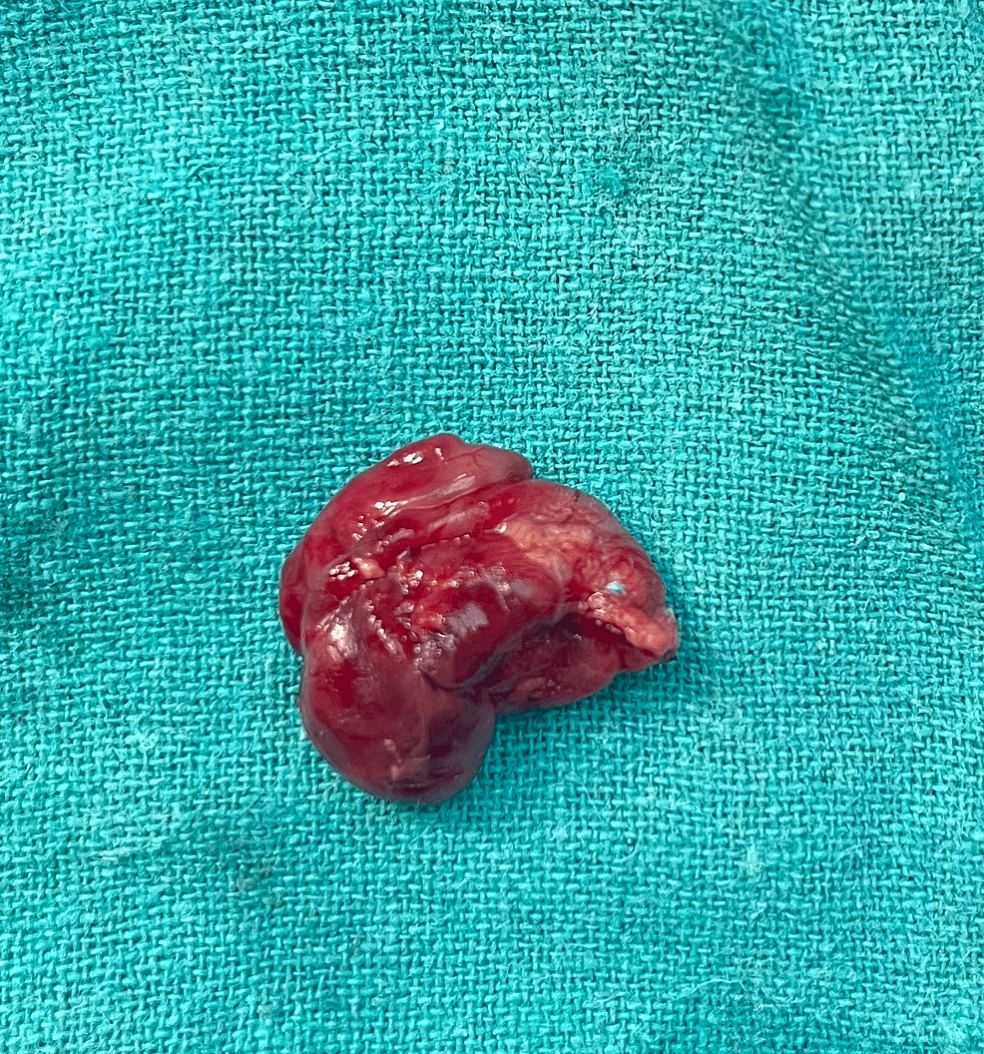
Excised swelling

The patient was planned for complete resection and excision of the swelling. A curvilinear pre-auricular incision was taken over the prominent part of the swelling. Skin flaps with parotid fascia were elevated, and swelling was identified (Figure 2). It was found to be cystic in consistency. This swelling was separated from surrounding structures, and after a thorough and meticulous dissection(Figure 3), the swelling was excised (Figure 4), preserving the facial nerve, and sent for histopathology. The wound was closed in layers. 

**Figure 5:**
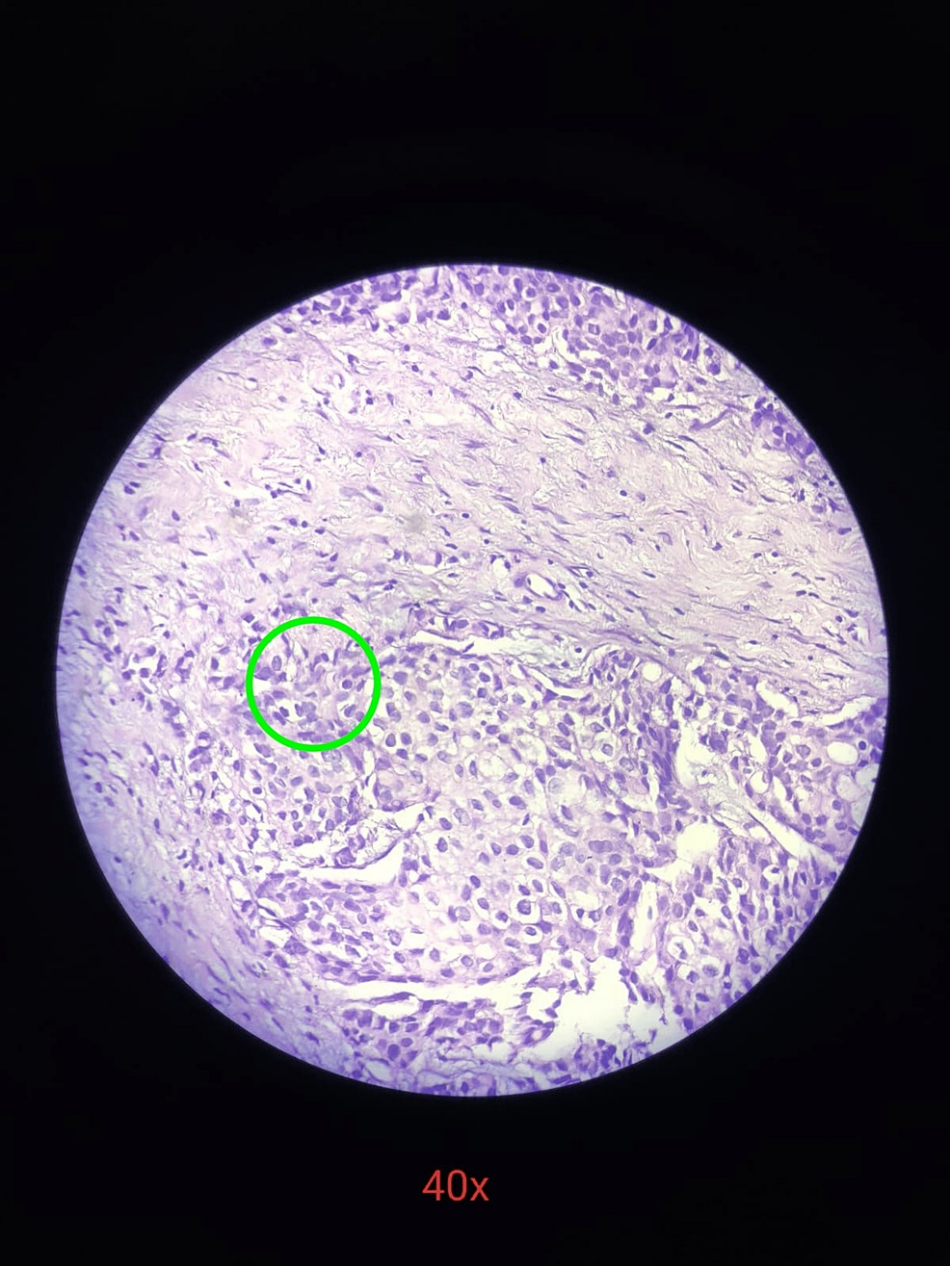
Histopathology slide under 40x magnification showing lymphocytic inflammatory infiltrate (green circle)

**Figure 6:**
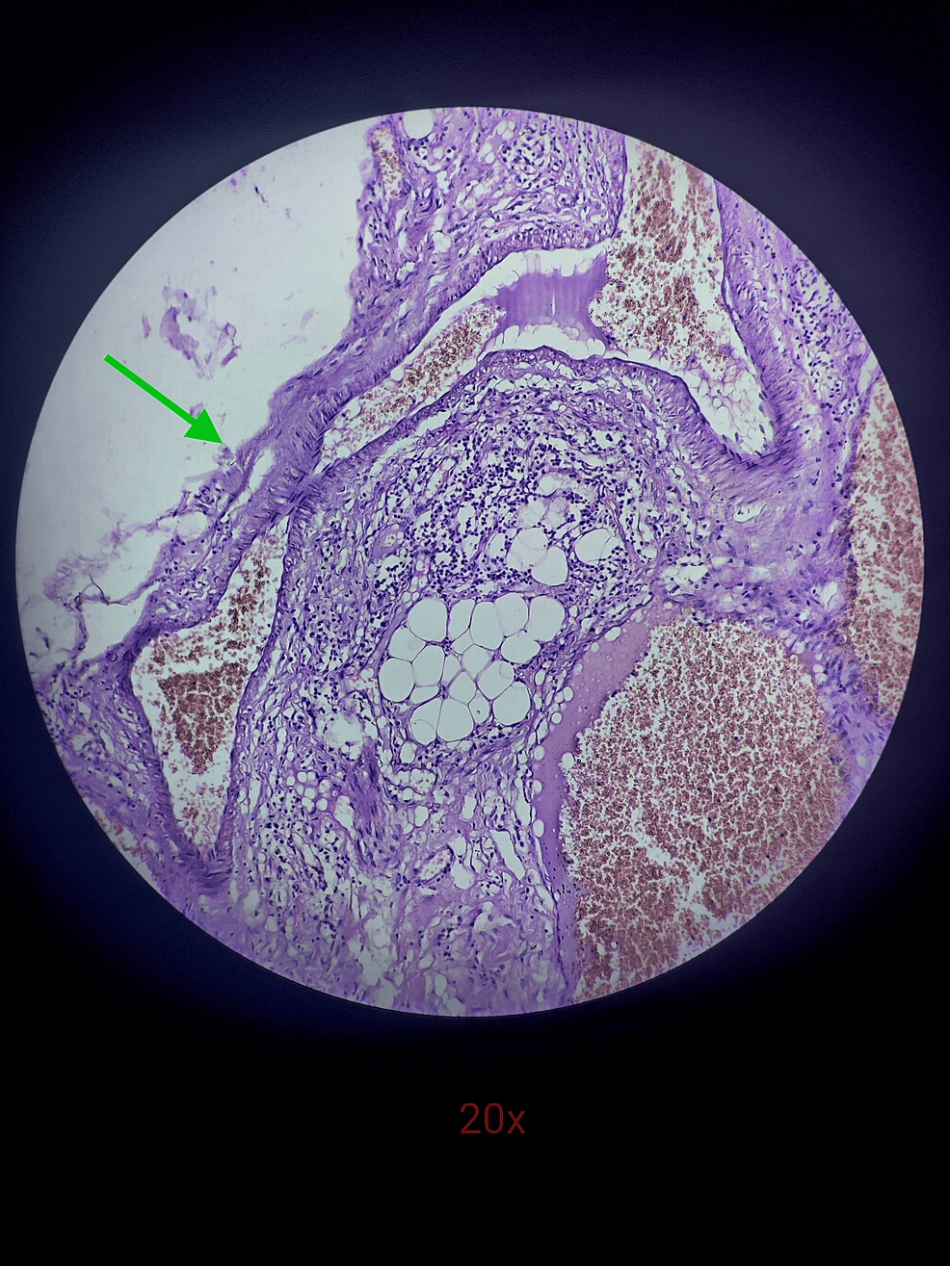
Histopathology slide image under 20x magnification showing lymphocyte infiltration in the epithelial lining with degenerative changes of the lining epithelium (green arrow)

**Figure 7:**
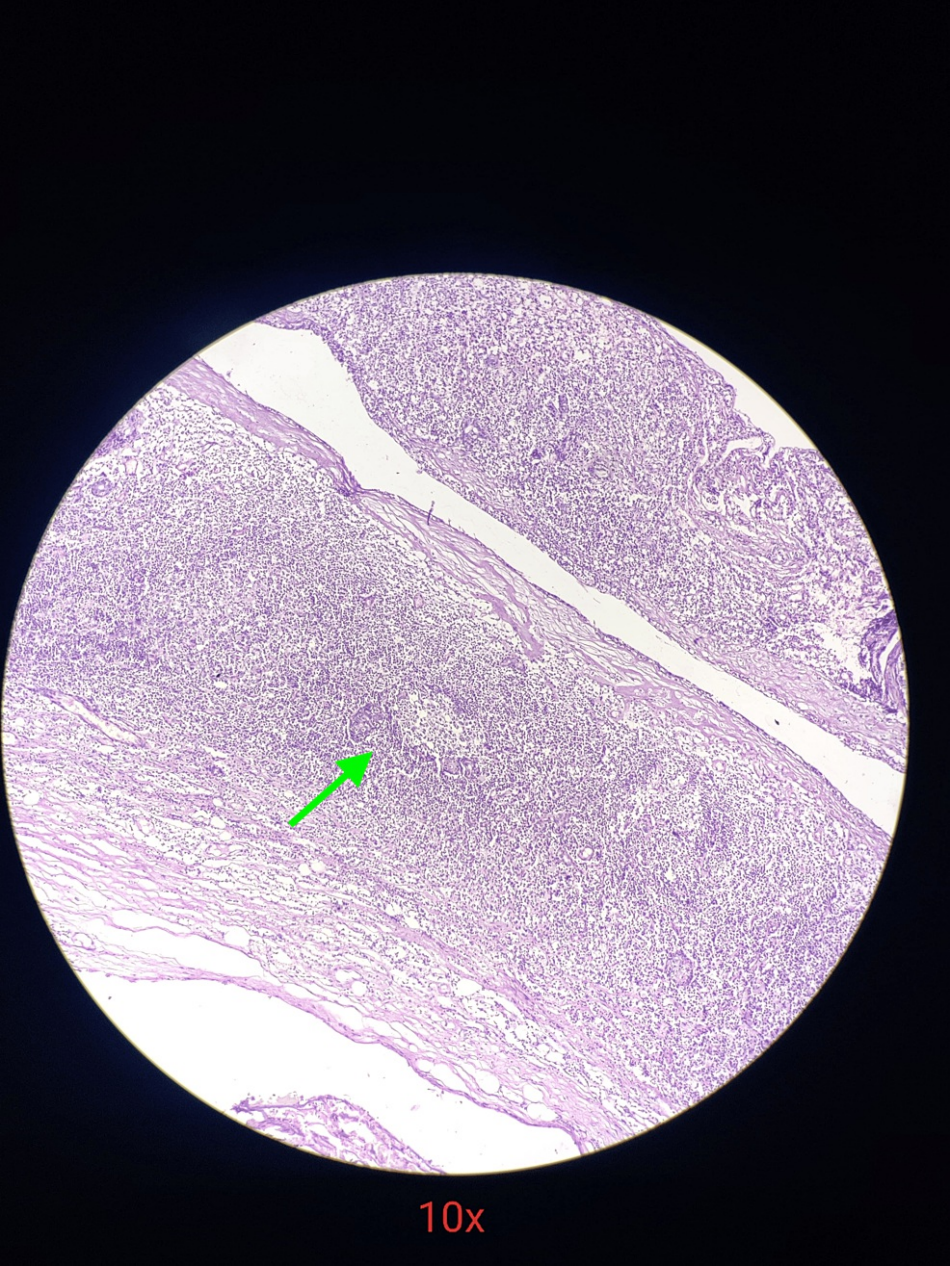
Histopathology slide image under 10x magnification showing polymorphous lymphoid cells (green arrow)

The postoperative period was uneventful. The patient was eventually discharged after suture removal and was kept on regular follow-up with the continuation of highly active antiretroviral therapy (HAART). The patient was started on tab dolutegravir, lamivudine, and tenofovir disoproxil fumarate combination (50mg/300mg/300mg) daily. She did not develop any signs of nerve palsy post-procedure. Histopathological slides showed cystic tissue lined by epithelium, which was attenuated at most places, and stratified squamous epithelium was visible. Cyst wall showing polymorphous lymphocytic infiltrates forming polymorphs. Epithelial cysts were seen in lymphoid areas on histopathology. Subepithelial stroma with reactive lymphoid follicles and germinal centres were identified on microscopic examination. The fatty tissue and nearby normal salivary glands were unimpaired, and neither neoplastic nor oncocytic cell lining was seen (Figures 5-7). Histopathological features were consistent with lymphoepithelial cysts.

Case two

Previously in 2012, an eight-year-old female patient presented with similar complaints of bilateral parotid swellings for six years with an associated history of fever, diarrhoea, and bilateral cervical and axillary lymphadenopathy. All routine investigations and subsequent serological testing were done, CD4/CD8 count was 697/1714 (Ratio: 0.41). Rapid HIV, ELISA, and Western blot tests were positive.

**Figure 8:**
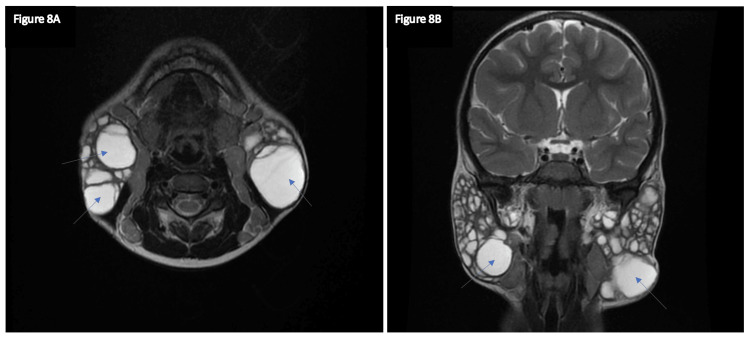
MRI Images in Axial view (A) and Coronal view (B) showing diffuse enlargement of parotid glands (blue arrows) suggestive of benign lymphoepithelial lesions

Cytological examination of the parotid swellings showed lymphocytes with a few polymorphs. Fine needle aspiration cytology was done, suggesting a lymphoproliferative disorder. The above history and clinical investigations confirmed the diagnosis of bilateral parotid lymphoepithelial cysts associated with DILS. An MRI scan was done, suggesting benign lymphoepithelial lesions (Figures 8A, 8B). The patient was medically managed, put on highly active antiretroviral therapy, and discharged once symptomatically better. No surgical intervention was done for this patient. She was kept on follow-up; after two months, the swelling regressed.

## Discussion

As evidenced by a case report submitted by Liao et al., even non-HIV-infected patients can develop parotid swellings associated with DILS. However, treatment modalities remain the same. In their case, surgical excision and complete resection of swelling were done, and the patient did not show any signs of recurrence post-surgery during two years of follow-up [3]. There are various treatment options available in the management of lymphoepithelial cysts. Some of these include medical management and close observation, repeated aspiration from the swelling site, anti-retroviral therapy, radiotherapy, sclerotherapy, and surgical excision.

Close observation with immunosuppressants like corticosteroids might temporarily regress enlarged tissues, but swelling can return with tapering these medications. Hence, It is now being prescribed in combination with anti-retroviral drugs for regression of the swellings. The use of anti-retroviral medication has been associated with a significant degree of regression of parotid swellings with time. Drugs like zidovudine (AZT) or, in our case, a combination of dolutegravir, lamivudine, and tenofovir disoproxil fumarate have shown potential in reducing or eliminating these lesions. In our second case, the patient was started on anti-retroviral drugs, and her symptoms and swellings gradually decreased in size over time. There was no need for further surgical intervention.

The use of sclerotherapy has demonstrated promise in reducing these cysts. Studies have shown a reduction in cyst size ranging from 42% to even 100% in some cases. While this process may result in slight oedema and tenderness, it is important to note that no severe complications, such as facial nerve injury or infection, have been reported [4,8]. Aspiration from swelling can be performed quickly. However, one drawback is recurrence, and most aspirated lesions tend to recur within time and continue to grow. Procedures of this nature should be contemplated for severely immunodeficient patients where the potential risks outweigh the benefits of surgical management. Radiation therapy is another treatment option that has shown promise in reducing the size of the parotid gland. However, it should generally be avoided due to concerns regarding malignant transformation [9].

Surgical excision is rarely recommended nowadays due to complications such as facial nerve injury intra-operatively or multiple surgical procedures due to recurrence, even after a superficial parotidectomy. These complications can be avoided by meticulous dissection during surgery. Statistically, there is a risk of 2-7% of complications during or post-surgical resection. Therefore, surgery is typically considered a last resort on the patient’s demand when all other treatment options have failed.

## Conclusions

Patients presenting with bilateral parotid swellings will usually correlate with a positive retro-viral status, and a thorough serological investigation is necessary to confirm this status. Over time, neither patient showed any signs of recurrence after completing our line of management; if the swellings are small in size and not cosmetically evident, the patient can be managed conservatively with HAART. However, if the swellings are large and prominent, leading to cosmetic discomfort, surgical excision is preferable, and care should be taken during surgery to avoid complications.
